# Supplementary material for: Integrating genomic structural equation modeling and experimental validation to unravel the genetic basis of male genital lichen sclerosus
Source: Front Immunol. 2026 Jul 16;17:1857945. doi: 10.3389/fimmu.2026.1857945 (PMC13435250; doi:10.3389/fimmu.2026.1857945)
Supplement: Supplementary file 6 [file SupplementaryFile5.zip › result/fuma_PSYCH_factor_sCCA2_focus.chr8.chr8.15991660.17387876.pop1.pdf]

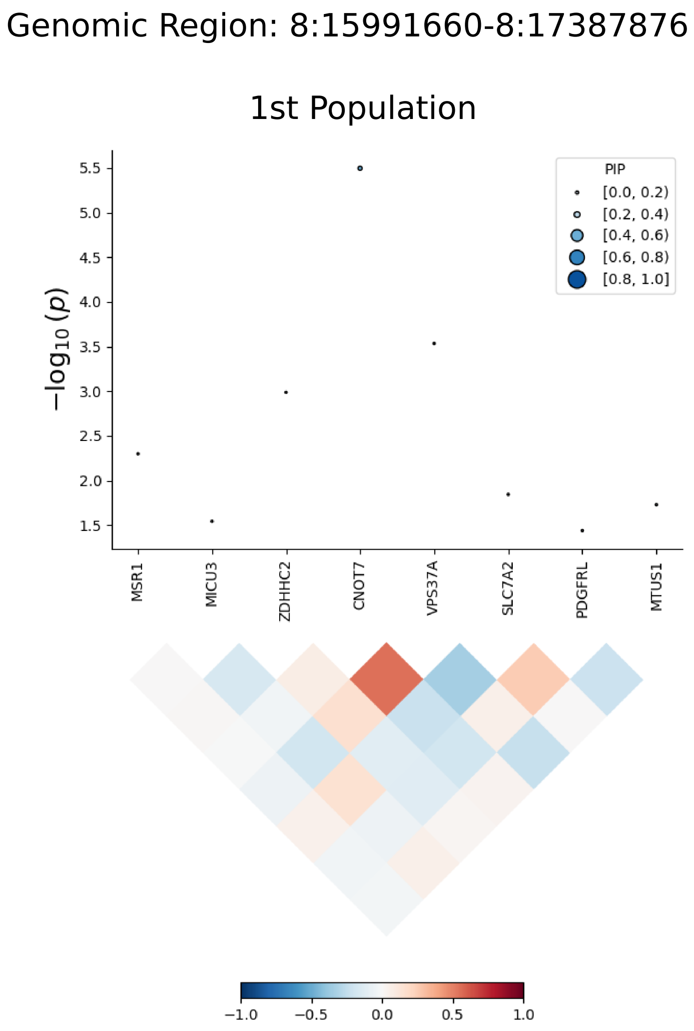

Genomic Region: 8:15991660-8:17387876
1st Population
$-\log_{10}(p)$
5.5
5.0
4.5
4.0
3.5
3.0
2.5
2.0
1.5
PIP
[0.0, 0.2)
[0.2, 0.4)
[0.4, 0.6)
[0.6, 0.8)
[0.8, 1.0]
MSR1
MICU3
ZDHHC2
CNOT7
VPS37A
SLC7A2
PDGFRL
MTUS1
−1.0
−0.5
0.0
0.5
1.0
